# Synergistic Enhancement of Cancer Therapy Using a Combination of Ceramide and Docetaxel

**DOI:** 10.3390/ijms15034201

**Published:** 2014-03-10

**Authors:** Li-Xia Feng, Min Li, Yong-Jun Liu, Shao-Mei Yang, Na Zhang

**Affiliations:** Department of Pharmaceutics, College of Pharmacy, Shandong University, 44 West Culture Road, Ji’nan 250012, China; E-Mails: fenglixia0910@126.com (L.-X.F.); sdlimin@sina.cn (M.L.); o-oliuyongjun@163.com (Y.-J.L.); yangshaomei01@163.com (S.-M.Y.)

**Keywords:** ceramide, docetaxel, combination therapy, anti-cancer, synergy mechanisms

## Abstract

Ceramide (CE)-based combination therapy (CE combination) as a novel therapeutic strategy has attracted great attention in the field of anti-cancer therapy. The principal purposes of this study were to investigate the synergistic effect of CE in combination with docetaxel (DTX) (CE + DTX) and to explore the synergy mechanisms of CE + DTX. The 3-(4,5-Dimethylthiazol-2-yl)-2,5-diphenyltetrazolium bromide (MTT) and combination index (CI) assay showed that simultaneous administration of CE and DTX with a molar ratio of 0.5:1 could generate the optimal synergistic effect on murine malignant melanoma cell (B16, CI = 0.31) and human breast carcinoma cell (MCF-7, CI = 0.48). The apoptosis, cell cycle, and cytoskeleton destruction study demonstrated that CE could target and destruct the microfilament actin, subsequently activate Caspase-3 and induce apoptosis. Meanwhile, DTX could target and disrupt the microtubules cytoskeleton, leading to a high proportion of cancer cells in G2/M-phase arrest. Moreover, CE plus DTX could cause a synergistic destruction of cytoskeleton, which resulted in a significantly higher apoptosis and a significantly higher arrest in G2/M arrest comparing with either agent alone (*p* < 0.01). The *in vivo* antitumor study evaluated in B16 tumor-bearing mice also validated the synergistic effects. All these results suggested that CE could enhance the antitumor activity of DTX in a synergistic manner, which suggest promising application prospects of CE + DTX combination treatment.

## 1. Introduction

Cancer is the leading cause of deaths worldwide and chemotherapy is one of the major trends on cancer therapy [1,2]. However, the problems of mono-chemotherapy, such as inadequacy of efficacy, drug resistance and systemic toxicity, would not be completely solved in a short time. Moreover, the administration doses of cytotoxic drugs cannot be limitlessly increased [3]. Based on this situation, combination therapy arose in response and was expected to achieve high therapeutic efficacy at lower drug dose [4]. Among various kinds of combination therapy strategies, combinations of multiple cytotoxic drugs have attracted significant attention [3]. For example, bevacizumab/gemcitabine on ovarian cancer [5], gemcitabine/taxane on metastatic breast cancer [6,7], and cisplatin/taxane [8] and cisplatin/gemcitabine [9] on non-small cell lung cancer have been widely studied and used. In spite of moderate increase in the therapeutic efficacy, combining multiple cytotoxic drugs for cancer therapy might simultaneously lead to a potential danger of drug interaction toxicity [10], cross-resistance [11] and a compromise in quality of life. Therefore, it is urgent to develop a novel combination therapy strategy for cancer treatment.

When evaluated in preclinical studies, some biologic agents plus cytotoxic drugs exhibit additive or synergistic activity without excessive toxicity, providing a promising direction for combination therapy [12]. For example, interleukin-2 (IL-2) and/or alpha-interferon (α-IFN) have been widely used in clinical study to combine with cisplatin, vinblastine, or 5-fluorouracil for the patient with metastatic cancer, which resulted in a synergistic therapeutic efficacy [13]. Among different biologic agents, ceramide (CE) showed great potential in designing novel combination therapy strategies [14]. CE is a naturally occurring sphingolipid, which is derived intracellularly by hydrolysis of the lipid sphingomyelin or by *de novo* synthesis through *N*-acylation of sphinganine [15,16]. CE has been newly realized as important intracellular signaling molecules that mediate diverse cellular effects, of which programmed cell growth, differentiation, and death have attracted significant interests in recent years [17,18]. Although the exact mechanism has not fully elucidated, CE has been identified as a putative anti-cancer therapeutic agent from a therapeutic perspective due to its important role in apoptosis [19]. Considering endogenous CE can subsequently be further metabolized by the enzyme glucosylceramide synthase (GCS) to yield glucosylceramide (GC) which does not have any proapoptotic activities and oppositely induce the serious multi-drug resistance (MDR), exogenous CE was introduced to preclinical study of cancer treatment [20]. It has been reported that, a polymeric nanoparticles, based on poly(ethylene oxide)-poly(epsilon caprolactone) (PEO-PCL), was prepared to co-deliver exogenous CE and Paclitaxel (PTX) [21]. Against a PTX-resistant ovarian cancer cell (SKOV-3TR), the combination of CE with PTX was found to raise PTX sensitivity of the MDR cells to the same level as non-MDR cells, which showed a 100-fold increase in therapeutic efficacy as compared to PTX alone. These exciting results showed great potential for combination of exogenous CE and cytotoxic drugs.

As this approach gathers more attention, it is becoming important to consider clinical issues such as choice of cytotoxic drugs, optimized dosing schedule, and mechanisms of synergy in order to identify the most effective combination treatment regimen [22,23]. Despite the supportive preclinical data, it is still unknown whether exogenous CE could be combined with different cytotoxic drugs to show synergistic therapeutic effects on various cancer treatments because of the heterogeneity of different cancers and the diverse anti-cancer mechanisms of different antineoplastic drugs. Therefore, there is an urgent need to investigate and develop a comparatively comprehensive strategy on CE-based combination therapy (CE combination).

In present study, the *in vitro* anti-proliferation effects of exogenous CE combining with three traditionally and widely used anti-cancer drugs: docetaxel (DTX), PTX, and Doxorubicin (DOX) were correspondingly evaluated on four different cancer cell lines: murine malignant melanoma cell line (B16), human breast carcinoma cell line (MCF-7), human ovarian carcinoma cell line (SKOV3), and human hepatocellular carcinoma cell line (HepG2), respectively, by 3-(4,5-Dimethylthiazol-2-yl)-2,5-diphenyltetrazolium bromide (MTT) assay. Then the combination index (CI) was further calculated to analyze whether there is a synergistic effect between CE and the chosen anti-cancer drug. To optimize the dosing schedule, the experiments of screening for optimal combination ratio and sequence of administration were subsequently carried out by MTT assay and CI assay. Cell apoptosis induction, Caspase-3 activity, cell cycle arrest and cytoskeleton destruction were systematically studied to exploit the mechanisms of synergy between CE and DTX. In order to verify the *in vivo* synergy effects, the in vivo antitumor efficacy of CE + DTX was also experimented.

## 2. Results

### 2.1. Effects of CE Combination on Cell Proliferation (MTT Assay)

The anti-proliferation effects of CE combination (CE + DTX, CE + PTX, or CE + DOX) at molar ratio 1:1 was evaluated at various concentrations by MTT assay on B16, SKOV3, MCF-7, and HepG2 cells, respectively.

**Figure 1.**
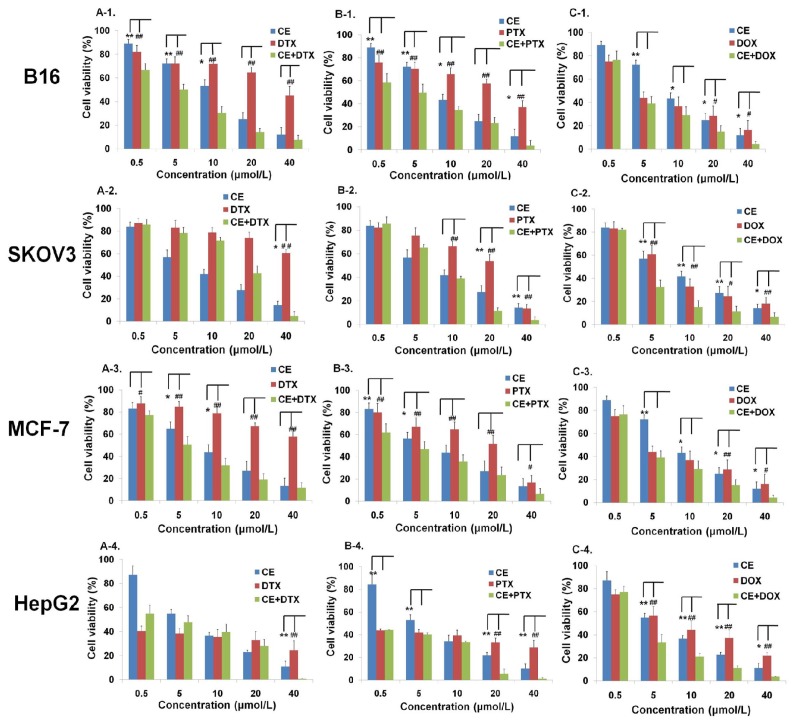
Effects of different treatments on cell viabilities (% from untreated control) of B16, SKOV3, MCF-7 and HepG2 cells (*n* = 3). Cells were treated with CE, DTX, PTX, DOX, or 1:1 combination molar ratio of CE plus one of the three anti-tumor drugs, respectively, at a series of concentration from 0.5 to 40 μM. (**A-1**–**A-4**) The comparative test among CE, DTX, and CE + DTX; (**B-1**–**B-4**) The comparative test among CE, PTX and CE + PTX; (**C-1**–**C-4**) The comparative test among CE, DOX and CE + DOX. * *p* < 0.05, ** *p* < 0.01, statistically significant difference between CE and combination treatment (CE + DTX, CE + PTX, or CE + DOX, respectively); ^#^ *p* < 0.05, ^##^ *p* < 0.01, statistically significant difference between DTX and combination treatment (CE + DTX, CE + PTX, or CE + DOX, respectively).

The results of the cell viability with different treatments were shown in Figure 1. In the case of B16 and MCF-7 cells, comparing with CE, DTX, PTX, or DOX, CE combination (CE + DTX, CE + PTX and CE + DOX) showed much lower cell viabilities at all given concentrations (except 0.5 μM), respectively, indicating a strong potential for combination treatment. For SKOV3 cells, only CE + DOX (5–40 μM) showed cytotoxicity enhancements compared with either agent alone (*p* < 0.05). In addition, CE + DOX, CE + PTX, but not CE + DTX, generated significantly higher anti-proliferation effects on HepG2 cells (*p* < 0.05) at some of the experimented concentrations.

**Figure 2.**
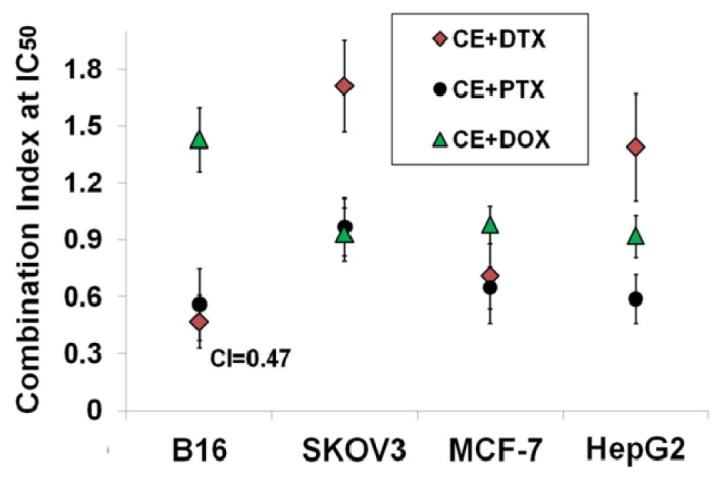
Combination index (CI) values of CE + DTX, CE + PTX, and CE + DOX at 50% growth inhibition point on B16, SKOV3, MCF-7, and HepG2 cells (*n* = 3).

In order to qualitatively evaluate whether the combination of CE with DTX, PTX, or DOX could generate synergistic antiproliferative effects, CI, a commonly used evaluation index, was calculated [24–26]. CI values at 50% growth inhibition points were calculated based on the results of MTT tests and the CI values were shown in Figure 2. CI values of CE + DTX were 0.47 on B16 cells and 0.71 on MCF-7 cells, respectively, indicating that the synergistic antiproliferative effect of CE + DTX was preliminarily established on B16 and MCF-7 cells. Similar synergistic effect was observed in CE + PTX combination treatment, with the CI values were 0.54 on B16 cells, 0.63 on MCF-7 cells and 0.55 on HepG2 cells, respectively, indicating that the combination of CE with PTX might also be promising. Meanwhile, no obvious synergy was found for CE + DOX and even slight antagonism was observed on B16 cells, which warned that the combination of CE with DOX might not be an optimal option for cancer treatment under such given conditions.

In comparison with the other anti-cancer drugs, DTX was most synergistic with CE on B16 cells for the CI value at 50% growth inhibition point was lowest (CI = 0.47, Figure 2), therefore, CE + DTX was chosen for further study. Correspondingly, the positive cell lines (cells showed synergistic antiproliferative effect): B16 and MCF-7 were chosen as model cells.

### 2.2. Determination of the Optimal Dose Schedule of CE + DTX on B16 and MCF-7 Cells

There is a need for optimizing the combination dose schedule of CE + DTX to realize its full therapeutic potential. It has previously been recognized that whether or not two agents interact synergistically or antagonistically is dependent on the ratio of the agents [27]. Moreover, the sequence of administration is particularly important since a given sequence might have an adverse result, such as antagonism instead of the required synergism [4]. Therefore, the experiments for screening of CE + DTX with optimal combination ratio and most suitable sequence of administration were carried out.

To determine the optimal combination ratio of CE and DTX, the anti-proliferation effects of CE + DTX with different combination ratio were tested by MTT assay and the corresponding CI values were calculated, respectively. As shown in Table 1, the CI values changed greatly (0.31–1.08 on B16 cells and 0.48–1.17 on MCF-7 cells, respectively) along with the variation of the combination molar ratio, which proved the importance of combination ratio in combination therapy. When CE and DTX combined with a molar ratio of 0.5:1, the lowest CI values were obtained on B16 (CI = 0.31) and MCF-7 cells (CI = 0.48), respectively. Consequently, the combination molar ratio of CE and DTX was determined as 0.5:1. For the screening of sequence of administration, CE or DTX was added sequentially and the cell viability was measured. Unexpectedly, there was no significant difference on the anti-proliferation effects between different sequential treatments (*p* > 0.05). Therefore, the sequence of administration was considered to have no obvious effect on the synergistic effect of CE + DTX in the present study. Taken together, simultaneous administration of CE and DTX with a molar ratio of 0.5:1 was finally determined as the optimal dose schedule for later studies.

### 2.3. Induction of Apoptosis on B16 and MCF-7 Cells by CE + DTX

**Figure 3.**
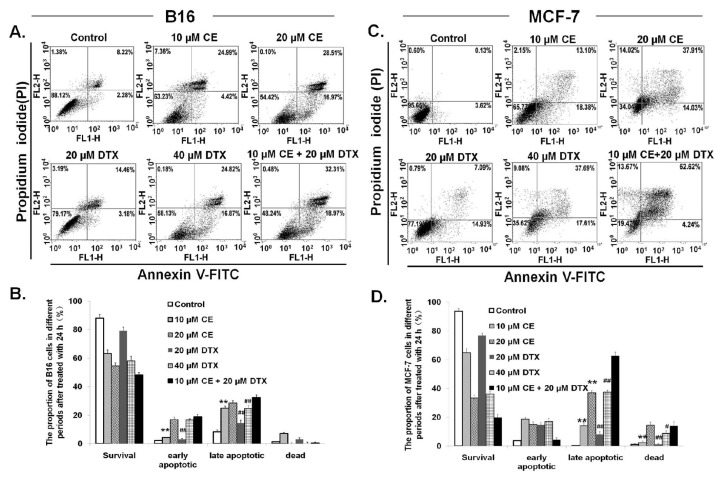
Induction of apoptosis caused by CE + DTX on B16 and MCF-7 cells (*n* = 3). Cells were treated with either CE (10 or 20 μM), DTX (20 or 40 μM), or CE + DTX (10 μM CE plus 20 μM DTX) for 24 h incubation, while 0.3% DMSO-PBS served as control. (**A**,**C**) Representative dot-plots illustrating apoptotic status on B16 and MCF-7 cells; (**B**,**D**) Data summary and analysis of the proportion of B16 and MCF-7 cells in different periods was according to the results of flow cytometric analysis. ** *p* < 0.01, statistically significant difference between CE and CE + DTX; ^#^ *p* < 0.05, ^##^ *p* < 0.01, statistically significant difference between DTX and CE + DTX.

To verify the synergistic effects of CE + DTX on B16 and MCF-7 cells, the apoptotic effects of CE + DTX were tested using Annexin V-FITC and PI apoptosis kit. The representative dot-plots illustrating apoptotic status were shown in Figure 3. In the case of B16 cells, the percentage of apoptotic cells (early apoptotic plus late apoptotic cells) treated with CE + DTX combination solution was 51.28% ± 5.16%, which was significantly higher in comparison with CE (29.41% ± 3.19%, *p* < 0.01) or DTX (17.64% ± 4.32%, *p* < 0.01), respectively. Furthermore, significantly higher proportion of late apoptosis caused by CE + DTX (32.31% ± 4.47%) was found compared with that of double-concentration DTX (24.82% ± 2.79%, *p* < 0.01), as shown in Figure 3B. For MCF-7 cells, similar results were observed (Figure 3C,D). These results indicated the potential synergistic enhancement of cancer therapy using CE + DTX. Furthermore, lower concentration of CE + DTX was able to induce a significantly higher apoptosis in comparison with double-concentration of DTX and CE, which would provide some enlightenment on that CE + DTX combination treatment might provide a promising prospect on toxicity reduction of chemotherapy.

### 2.4. Activation of Caspase-3 on B16 and MCF-7 Cells by CE + DTX

**Figure 4.**
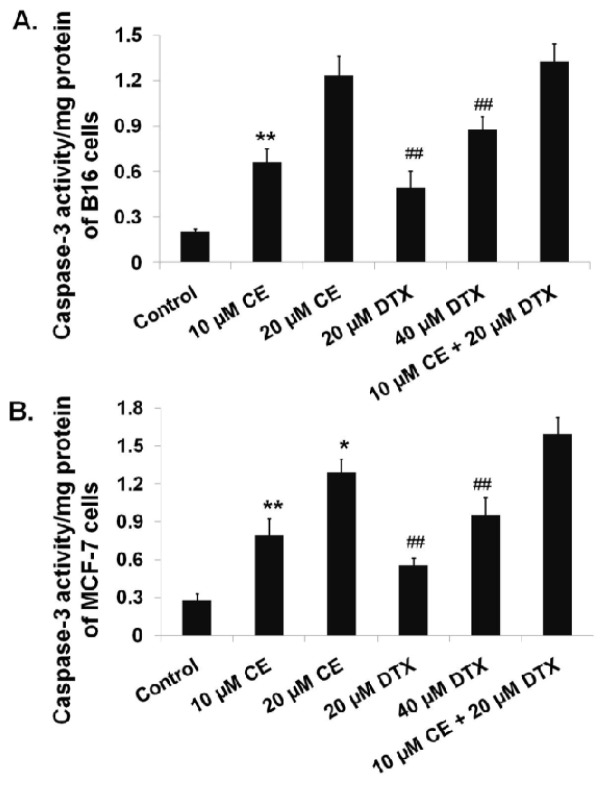
Significant enhancement of Caspase-3 activity caused by CE + DTX on B16 and MCF-7 cells (*n* = 3). Cells were treated with either CE (10 or 20 μM), DTX (20 or 40 μM), or CE + DTX (10 μM CE plus 20 μM DTX) for 24 h incubation, while 0.3% DMSO-PBS served as control. The Caspase-3 activity of cells with different treatments was evaluated by assessing the capacity to catalyze the cleavage of Caspase-3 substrate (Ac-DEVD-pNA) and release the pNA fluorochrome. (**A**) Caspase-3 activity of B16 cells; (**B**) Caspase-3 activity of MCF-7 cells. * *p* < 0.05, ** *p* < 0.01, statistically significant difference between CE and CE + DTX; ^##^ *p* < 0.01, statistically significant difference between DTX and CE + DTX.

Caspases-3 is one of the crucial mediators of apoptosis, being essential for certain processes associated with the dismantling of the cell and the formation of apoptotic bodies [28]. To confirm the probable pathways of synergistic apoptotic effect of CE + DTX, Caspase-3 activities of B16 and MCF-7 cells were evaluated by a Caspase-3 Activity Assay Kit. As shown in Figure 4, significantly higher Caspase-3 activity of B16 cells was observed upon cells treated with CE + DTX (10 μM CE plus 20 μM DTX) comparing with that of cells treated with DTX (20 μM), DTX (40 μM), or CE (10 μM) (*p* < 0.01), respectively. For MCF-7 cells, the Caspase-3 activity activated by CE + DTX was significantly higher than that of cells treated with DTX (20 μM), DTX (40 μM), or CE (10 μM) (*p* < 0.01), respectively, and even higher than that of cells treated with 20 μM CE (*p* < 0.05). These results were consistent with the study of apoptosis tests. Taking the aforementioned results together, it was reasonable to believe that the synergistic enhancement of apoptosis caused by CE + DTX was probably mediated by the significant activation of Caspase-3.

### 2.5. Cell Cycle Effect of CE + DTX

**Figure 5.**
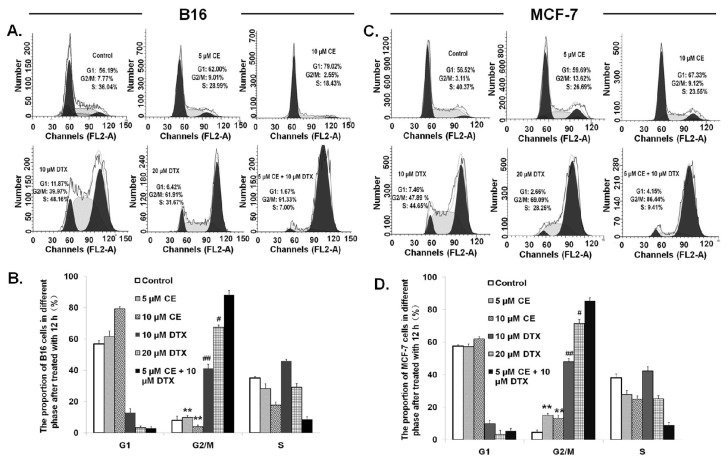
Cell cycle effect of CE + DTX on B16 and MCF-7 cells (*n* = 3). Cells were treated with either CE (5 or 10 μM) or DTX (10 or 20 μM), or combination (5 μM CE plus 10 μM DTX) for 12 h incubation, while 0.3% DMSO-PBS served as control. (**A**,**C**) Representative experiments on B16 and MCF-7 cells, respectively; (**B**,**D**) Data summary and analysis of the proportion of B16 cells and MCF-7 cells in different phase after treatment according to the results of flow cytometric analysis. ** *p* < 0.01, statistically significant difference between CE and CE + DTX; ^#^ *p* < 0.05, ^##^ *p* < 0.01, statistically significant difference between DTX and CE + DTX.

It has been intensively studied that cells have a higher frequency of arrest in G2/M phase by DTX [29], however, there is no published data to report the effect of CE on cell cycle progression. To better understand the mechanisms responsible for the synergistic anti-proliferative activity of CE + DTX, the cell cycle distribution of B16 and MCF-7 cells was evaluated by flow cytometric analysis. As shown in Figure 5, cells were arrested in G1 phase by CE while arrested in G2/M phase by DTX, both in dose-dependent manner. B16 cells were arrested in G2/M-phase (91.33%) by CE + DTX, that was significantly higher in comparison with that of either agent alone (*p* < 0.01), with an accompanying decrease in the G1 phase or S phase. Moreover, the level of G2/M accumulation caused by CE + DTX was even significantly higher than that caused by double-concentration CE (10 μM, *p* < 0.01) or DTX (20 μM, *p* < 0.05). Considering the single agent CE has no effect on G2/M arrest, it might be assumed that CE acted synergistically with DTX and consequently enhanced the therapeutic effect of DTX, which contributed to the synergistic arrest in G2/M phase. In accord with B16 cells, the redistribution of cell cycle caused by CE + DTX also occurred in MCF-7 cells, as is shown in Figure 5C,D. These results corroborated the synergy between CE and DTX, indicating that CE + DTX produced greater potential therapeutic effects might also via the significant enhancement of G2/M-phase accumulation.

### 2.6. Cytoskeleton Destruction Effect of CE + DTX

Cytoskeleton is a dynamic structure essential for a wide variety of normal cellular processes, including the maintenance of cell shape and morphology, membrane dynamics, and signal transduction [30,31]. To explore whether CE and DTX have a synergistic effect on alteration of cytoskeleton, F-actin and β-tubulin of B16 and MCF-7 cells were specifically stained by FITC-phallacidin and Tubulin-Tracker Red, respectively, after treatment with CE + DTX (5 μM CE plus 10 μM DTX) for 12 h. The nuclei were stained by Hoechst 33342 in order to observe the cell morphology better.

**Figure 6.**
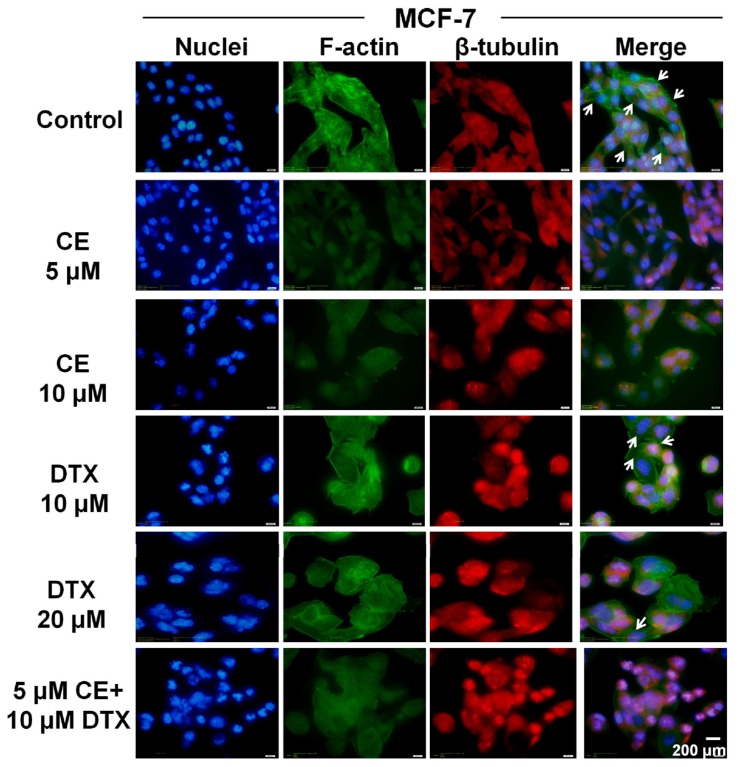
Cytoskeleton destruction effect of CE + DTX on MCF-7 cells (*n* = 3). The blue color indicated the location of nuclei, the green color indicated the location of F-actin and the red color represented the β-tubulin. The white arrows indicate the stress fibers of F-actin. Scale bar, 200 μm.

As shown in Figure 6, CE + DTX not only made a strong destruction on microfilaments and microtubules in comparison with either agent alone, but also seriously damaged the cell morphology, demonstrating a synergistic effect on destruction of cytoskeleton. DTX specifically promoted the assembly of β-tubulin but not F-actin and obviously induced the polymerization of microtubule, resulting in the enhancement of red fluorescence intensity. This was consistent with the literature and well coincide with the results of the G2/M-phase arrest tests [29]. Meanwhile, cells treated with CE showed no significant alteration on microtubule, however, the stress fibers of F-actin disappeared obviously, predicating the destruction of the microfilaments cytoskeleton. For B16 cells, similar results were observed (Figure S1).

**Figure 7.**
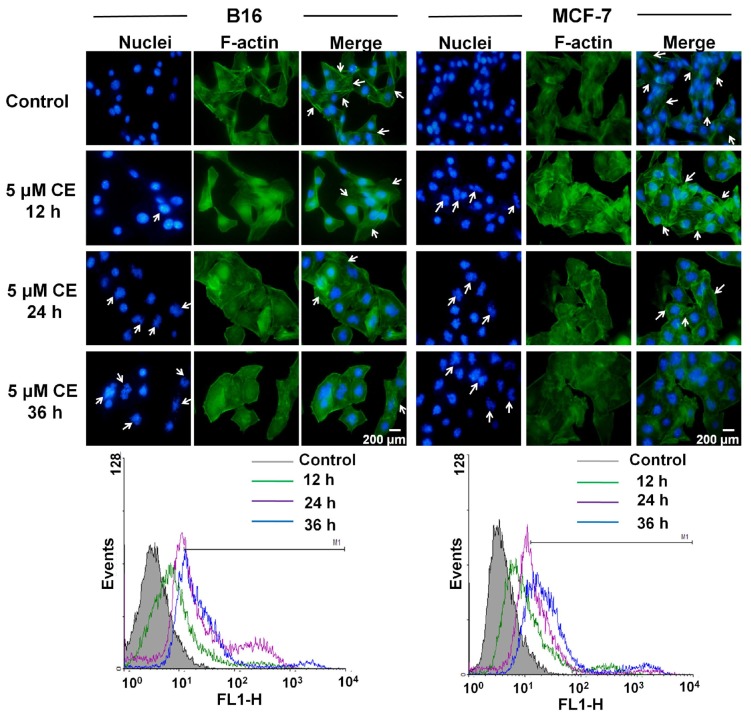
Alterations of microfilaments cytoskeleton caused by CE on B16 and MCF-7 cells (*n* = 3). The blue color indicated the location of nuclei and the green color indicated the location of F-actin. The white arrows indicate the formation of apoptosis body or stress fibers of F-actin. Scale bar, 200 μm.

To obtain a clear relationship between CE and microfilaments cytoskeleton, cells were incubated with 5 μM CE for 12, 24, and 36 h, respectively, the alterations of F-actin over time were observed. As shown in Figure 7, with prolongation of incubation time, the stress fibers of F-actin gradually disappeared and the polymerization of F-actin was significantly increased (*p* < 0.05). The flow cytometric results showed that B16 cells incubated with 5 μM CE for 12, 24, and 36 h showed 16.58% ± 5.58%, 51.21% ± 6.34%, and 69.52% ± 3.69% fluorescence positivity, respectively, while MCF-7 cells showed 38.40% ± 4.78%, 54.92% ± 5.70%, and 78.62% ± 4.45% fluorescence positivity, respectively. These results quantitatively proved the polymerization of F-actin and finally resulted in strong destruction of actin cytoskeleton. In addition, the destruction of actin cytoskeleton was accompanied by apoptosis as obvious fragmentation of nuclei and formation of apoptotic bodies were found after incubation with CE (Figure 7). Thus, it would be considered that CE could target and disrupt the actin cytoskeleton, resulting in the induction of apoptosis of cells subsequently. In conclusion, CE and DTX acted complementarily to destruct the cytoskeleton structure on B16 and MCF-7 cells, which could also lead to the significant enhancement of antitumor effect of CE + DTX.

### 2.7. In Vivo Anti-Tumor Effect of CE + DTX

**Figure 8.**
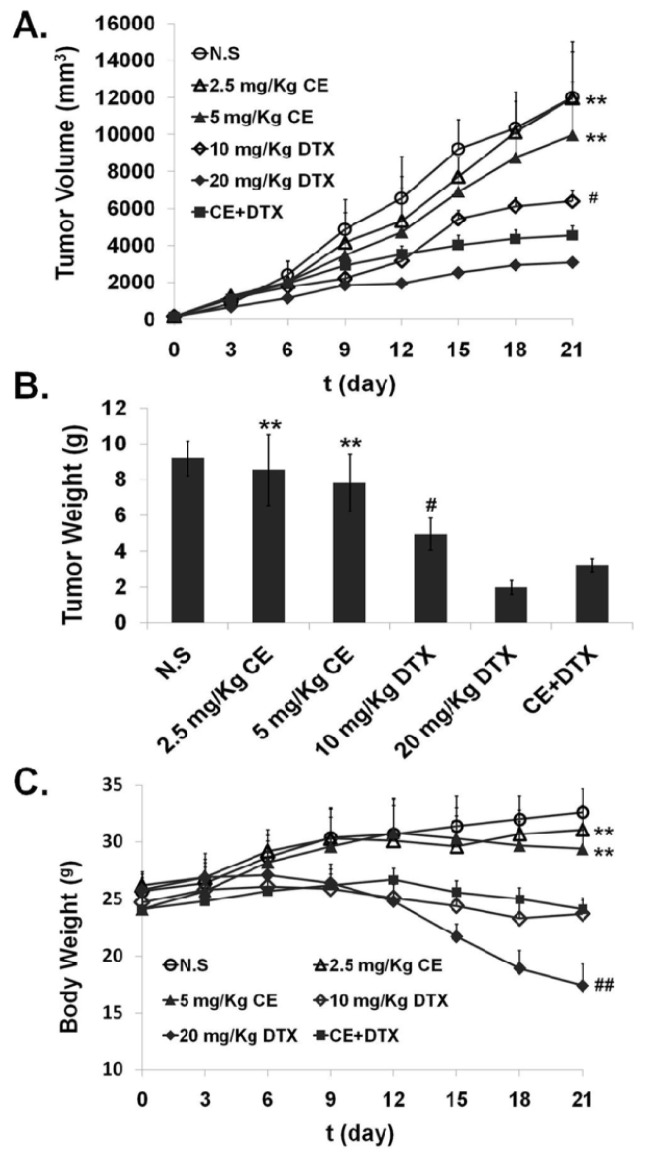
Antitumor effects of CE + DTX on B16 tumor-bearing mice after intravenous administration (*n* = 5). 2.5/5 mg/kg CE, 10/20 mg/kg DTX and N.S were served as controls. (**A**) Tumor volume; (**B**) Tumor weight; (**C**) Body weight. ** *p* < 0.01, statistically significant difference between CE and CE + DTX; ^#^ *p* < 0.05, ^##^ *p* < 0.01, statistically significant difference between DTX and CE + DTX.

Given that the synergistic effect of CE + DTX *in vitro*, it was essential to determine whether CE combined with DTX could enhance the antitumor activity *in vivo*. Therefore, the anti-tumor activity of CE + DTX (CE: 2.5 mg/kg, DTX: 10 mg/kg) was evaluated using B16 tumor-bearing mice, 2.5/5 mg/kg CE, 10/20 mg/kg DTX, and N.S were served as controls. The variation on tumor volume and body weight with different administration were monitored and recorded respectively, as shown in Figure 8.

Tumor volume is an important indicator for evaluating antitumor efficacy of the different therapy regimens [32]. As shown in Figure 8A, the antitumor efficacy of CE + DTX was significantly higher than that of single-dose of CE (2.5 mg/kg, *p* < 0.01) and DTX (10 mg/kg, *p* < 0.05), and even significantly higher than that of double dose of CE (5 mg/kg, *p* < 0.01), indicating the synergistic antitumor efficacy *in vivo*. To quantitatively evaluate the tumor regression effect of CE + DTX, the excised tumor of sacrificed mice were weighed. In accordance with the results of tumor volume, the tumor weight of mice after treatment with CE + DTX was 3.024 ± 0.36 g, which was significantly smaller than that of CE (2.5/5 mg/kg, *p* < 0.01) and DTX (10 mg/kg, *p* < 0.05) (Figure 8B), respectively.

On the other hand, severe systematic toxicity accompanying the high dose anti-cancer drugs is also a limiting factor for chemotherapy. The body weights of mice were monitored as an index of systemic toxicity [33] and the body weight variations of mice was shown in Figure 8C. During the experiment period, the weight loss induced by CE + DTX was significantly lower than that induced by DTX (20 mg/kg, *p* < 0.01), displaying a significantly lower systematic toxicity. The body weight of mice treated with CE + DTX was even higher than that of mice treated with 10 mg/kg DTX, which could verify our abovementioned opinion and further prove the safety of CE + DTX combination treatment. Although the body weight of mice treated with CE + DTX was lower comparing with that of N.S and 2.5/5 mg/kg CE, this phenomenon might be caused by the significant increase of tumor weight of the mice treated with N.S and 2.5/5 mg/kg CE (Figure 8A,B). Overall, these results indicated that CE combined with DTX could generate a synergistic antitumor efficacy *in vivo* and reduce the systematic toxicity in murine malignant melanoma model.

## 3. Discussion

Combination therapy plays a more and more important role in in the field of anti-cancer therapy and CE-based combination therapy as a novel therapeutic strategy has attracted great attention. To evaluate whether exogenous CE could in synergy with different anti-cancer drugs on various cancer cells, a comprehensive exploration of all possible combinations is ideal but cost-prohibitive and time-prohibitive. Based on these considerations, three traditionally and widely used anti-cancer drugs (DTX, PTX, and DOX) were selected as model drugs, correspondingly, the sensitive cell lines B16, MCF-7, SKOV3, and HepG2 were selected as model cells.

For preliminary screening, the combination molar ratio was fixed at 1:1 [34], therefore, the cytotoxicity of CE combination (CE + DTX, CE + PTX, or CE + DOX) at molar ratio 1:1 was evaluated at various concentrations by MTT assay on B16, MCF-7, SKOV3, and HepG2 cells, respectively. It was worth mentioning that, DTX and PTX should be expected to impose a large inhibitory growth response on these chosen cancer cells, however, the observed IC50 value for single agent DTX or PTX was large and higher than that of previous findings [35,36]. To verify these results, Taxol^®^ (Bristol-Myers Squibb, New York, NY, USA) and Duopafei^®^ were set as controls, finding that the cytotoxicity of Duopafei^®^ (Qilu Pharmaceutical Co., Ltd., Ji’nan, China) and Taxol^®^ to cells were high, as the IC50 values were much lower (Duopafei^®^: 2.31 μM on MCF-7 cells and Taxol^®^: 1.59 μM on B16 cells). It was indicated that the high cytotoxicity of Duopafei^®^ and Taxol^®^ were probably caused by Tween^®^-80-ethanol (Sinopharm Co., Ltd., Shanghai, China) and Cremophore EL^®^-ethanol (Sinopharm Co., Ltd., Shanghai, China), respectively.

CI assay, a commonly used evaluation methods to investigate whether there exists synergistic effects was developed from Chou [27]. In our study, simultaneous administration of CE and DTX at molar ratio of 0.5:1 showed highest synergistic effects on B16 and MCF-7 cells. Despite of some negative results, it is inappropriate to make an absolute or assertive conclusion that CE + DTX should be inhibited on SKOV3 cells or HepG2 cells (CI > 1.3, Figure 2). The exceptional results probably caused by the heterogeneity of different cancer cells and the diverse anti-cancer mechanisms of different drugs. Moreover, the irrational combination ratio of the combined agents or the inappropriate sequence of administration could also contribute to the negative results, which was also proved in Table 1. The CI test reminded that rational and effective designed strategies are important in combination therapy.

CE is one of the important intracellular signaling molecules, playing an important role in apoptosis [20], while DTX has been intensively studied to make cells have a higher frequency in G2/M arrest [29]. Based on these considerations, the apoptosis and cell cycle study was carried out. It was showed that, DTX could synergistically promote the apoptosis effect of CE, resulting in the significantly higher proportion of apoptosis cells (Figure 3). On the other hand, CE could act synergistically with DTX and consequently enhance the therapeutic effect of DTX, which contributed to the synergistic arrest in G2/M phase (Figure 5). Both the apoptosis and cell cycle study corroborated the synergy between CE and DTX.

As mentioned above, cytoskeleton is a dynamic structure essential for a wide variety of normal cellular processes, including the maintenance of cell shape and morphology, membrane dynamics and signal transduction [30,31]. Cytoskeleton could be organized into microtubules, microfilaments and intermediate filaments. Among them, microtubules have been extensively and clearly studied as targets of taxanes, such as Taxol and docetaxel, which have been used successfully clinically in treating various malignant diseases [37,38]. Microfilament actin is ubiquitous protein present in all eukaryotic cells and the disruption of F-actin has been found in malignant transformed cells [39]. Actin polymerization and remodeling play a critical role in the morphologic and phenotypic events in cancer cells [40]. Thus, it is reasonable to assume that microfilament actin, such as microtubules, could be a potential target for anti-cancer drug development [39,41].

In the present study we found that CE could target the microfilament actin, leading to the polymerization and destruction of actin cytoskeleton. As a result, Caspase-3 was activated [28] and subsequently induced apoptosis. The close relationship between apoptosis and Caspase-3 has also been proved in Figure 3. Meanwhile, DTX could target and disrupt the microtubules cytoskeleton, leading to a high proportion of cancer cells in G2/M-phase arrest. Moreover, CE plus DTX could cause a synergistic destruction of cytoskeleton, which resulted in a significantly higher apoptosis and a significantly higher arrest in G2/M arrest comparing with either agent alone (*p* < 0.01). Therefore, it is reasonable to deduce that the anti-tumor mechanisms of CE and DTX are probably complementary, which may contribute to a synergistic therapeutic effect of CE + DTX. The *in vivo* antitumor study also verified the synergy between CE and DTX, as CE + DTX could achieve a significantly higher antitumor efficacy than either agent alone (DTX, *p* < 0.05; CE, *p* < 0.01, Figure 8).

## 4. Experimental Section

### 4.1. Materials

Ceramide (CE) was obtained from Avanti Polar Lipids, Inc., Alabaster, AL, USA. Paclitaxel (PTX) and docetaxel (DTX) were provided by Chenxin Pharmaceutical Co., Ltd., Jining, China. Doxorubicin HCl (DOX) was purchased from Dalian Meilun Biology Technology Co., Ltd., (Dalian, China). 3-(4,5-Dimethylthiazol-2-yl)-2,5-diphenyltetrazoliumbromide (MTT) was purchased from Solarbio (Shanghai, China). Annexin V-FITC and propidium iodide (PI) apoptosis kit was provided by Bestbio (Shanghai, China). Caspase-3 Activity Assay Kit and Tubulin-Tracker Red were obtained from Beyotime (Shanghai, China). RNase A and PI were provided by TransGen Biotech Co., Ltd., (Beijing, China). FITC-Phallacidin and Hoechst 33342 were purchased from Invitrogen by Life Technologies (Carlsbad, CA, USA).

All the other chemicals and reagents used were of analytical purity grade or higher, obtained commercially.

### 4.2. Cell Lines and Cell Culture

Murine melanoma cell line (B16) and human breast carcinoma cell line (MCF-7) were purchased from the Chinese Academy of Sciences (Shanghai, China). Human ovarian carcinoma cell line (SKOV3) and human hepatocellular carcinoma cell line (HepG2) were kindly provided by Institute of Immunopharmacology and Immunotherapy of Shandong University (Ji’nan, China). HepG2 cell line was cultured in 25 cm^2^ culture flask in DMEM media, while B16, MCF-7 and SKOV3 cell lines were cultured in RPMI-1640 media at 37 °C under 5% CO_2_. All the media were supplemented with 10% (*v*/*v*) fetal bovine serum from Sijiqing Co., Ltd., (Hangzhou, China), streptomycin at 100 μg/mL and penicillin at 100 U/mL.

### 4.3. Animals

The female Kunming mice (weight: 18–22 g, age: 6–8 weeks) were supplied by the Medical Animal Test Center of Shandong University (Ji’nan, China). The animals were acclimatized for at least 48 h before experimentation, fed with a standard diet and allowed water *ad libitum*. All experiments were carried out in compliance with the Animal Management Rules of the Ministry of Health of the People’s Republic of China (document number 55, 2001) and the Animal Experiment Ethics Review of Shandong University.

### 4.4. Anti-Proliferation Test in Vitro

To investigate the anti-proliferation effects of CE-based combination treatment, an MTT assay was performed on B16, SKOV3, MCF-7, and HepG2 cells, respectively, following the manufacturer’s instructions. Briefly, cells in the exponential growth phase were counted and added into the 96-multiwell plate. Cells were allowed to adhere overnight at 37 °C and 5% CO_2_ before drug treatments. To prepare the stock solution, CE, PTX, and DTX were dissolved in dimethyl sulfoxide (DMSO) while DOX was dissolved in pH 7.4, 0.01 M phosphate-buffered saline (PBS). The stock solutions (CE solution, DTX solution, PTX solution or DOX solution) were serially diluted with serum supplemented media, making sure the final concentration of the drug was 0.5, 5, 10, 20, and 40 μM, respectively. To test the synergistic anti-proliferation effect of CE-based combination (CE plus DTX, CE plus PTX, or CE plus DOX), the corresponding combination solutions (CE + DTX, CE + PTX, and CE + DOX) were prepared proportionally at 1:1 molar ratio, respectively, making sure the final concentration of each agent was consistent with the above-mentioned concentration. Cells grown in the media containing an equivalent amount of DMSO without any drug served as control. The maximal concentration of DMSO in all the experimental wells was lower than 0.3% (*v*/*v*) [42]. After 48 h incubation, the cell viability was determined by absorbance measurements at 570 nm and a reference wavelength of 630 nm, measured by a microplate reader (FL600, Bio-Tek Inc., Winooski, VT, USA).

To optimize the dosing schedule, the synergistic effects of CE + DTX with different combination ratio and different sequence of administration were subsequently investigated (DTX was selected based on the results of the primary anti-proliferation tests mentioned above). To screen the optimal combination ratio, a series of concentration gradients of CE + DTX combination solutions were prepared proportionally at 0.25:1~4:1 molar ratio, and the anti-proliferation effects were studied by MTT method mentioned above. Based on the obtained optimal combination ratio, a series of concentration gradients of CE solution and DTX solution were prepared separately and administered in a chronological order (−4, −2, 0, 2, 4 h), to determine the optimal sequence of administration. All the other operations were consistent with the above-mentioned methods.

### 4.5. Calculation of Combination Index (CI)

The specific interaction between CE and DTX, PTX, or DOX on various cancer cell lines was evaluated by the combination index (CI) assay [24–26], respectively. The CI values were calculated based on the results of anti-proliferation tests and CI = D_1_/D_f1_ + D_2_/D_f2_ + D_1_D_2_/D_f1_D_f2_. Where D_f1_ is the concentration of Drug-1 required to produce x percent effect alone and D_1_ is the concentration of Drug-1 required to produce the same x percent effect in combination with Drug-2; Similarly, D_f2_ is the concentration of Drug-2 required to produce x percent effect alone and D_2_ is the dose of Drug-2 required to produce the same x percent effect in combination with Drug-1. Consequently, CI > 1.3: antagonism; CI 1.1–1.3: moderate antagonism; CI 0.9–1.1: additive effect; CI 0.8–0.9: slight synergism; CI 0.6–0.8: moderate synergism; CI 0.4–0.6: synergism; CI 0.2–0.4: strong synergism. In the present study, CI values at 50% growth inhibition effect were tested.

### 4.6. Induction of Apoptosis on B16 and MCF-7 Cells

To verify the synergistic effect of CE combination, the induction of apoptosis caused by CE + DTX on B16 and MCF-7 cells were carried out. Apoptosis detection was performed using the Annexin V-FITC and PI apoptosis kit (Bestbio, Shanghai, China). B16 and MCF-7 cells were plated at a density of 2 × 10^5^ cells/well into 12-well plates and incubated overnight. Apoptosis was induced by treating cells with CE + DTX combination solution (final concentrations of CE and DTX were 10 and 20 μM, respectively). Cells treated with CE solution (10 or 20 μM) and DTX solution (20 or 40 μM) were set at the same time to make a valid comparison against CE + DTX. Cells grown in media containing an equivalent amount of DMSO without any drug were served as control. After 24 h of incubation, the cells with different treatments were harvested and the final samples were measured on a FACS Calibur flow cytometry (BD Biosciences, Franklin Lakes, NJ, USA).

### 4.7. Evaluation of Caspase-3 Activity

In order to investigate whether CE + DTX would significantly activate Caspase-3 in comparison with CE or DTX, the Caspase-3 activity after different treatments on B16 and MCF-7 cells were evaluated by Caspase-3 Activity Assay Kit. Briefly, Caspase-3 was activated by treating cells with CE + DTX combination solution (final concentrations of CE and DTX were 10 and 20 μM, respectively). Cells treated with CE solution (10 or 20 μM) and DTX solution (20 or 40 μM) were set as controls. Cells grown in media containing an equivalent amount of DMSO without any drug were served as blank control. After 24 h incubation, the cells with different treatments were harvested and resuspended with 1× lysis buffer in the assay kit for 15 min at 0 °C, and then centrifuged at 20,000× *g* for 15 min to collect the supernatant containing cell extracts. To initiate the enzymatic reaction, the fresh cell extracts with different treatments were transferred into the well of a 96-well plate, respectively, followed by the addition of assay buffer and Caspase-3 substrate (Ac-DEVD-pNA). The absorbance was measured at 405 nm by a microplate reader (FL600, Bio-Tek Inc., Winooski, VT, USA), making sure the plate was maintained in dark at 37 °C during the measurements. In order to quantify the Caspase-3 activity, the protein content of each drug treatment was evaluated by Bradford protein assay (Beyotime, Shanghai, China), following the manufacturer’s assay instructions.

### 4.8. Cell Cycle Analysis of B16 and MCF-7 Cells

To study the effects of CE combination on cell cycle distribution, cell cycle analysis was assessed using flow cytometry. B16 and MCF-7 cells were exposed to CE + DTX combination solution (final concentration of CE and DTX were 5 and 10 μM, respectively) at 37 °C under 5% CO_2_, while cells treated with CE solution (5 or 10 μM) and DTX solution (10 or 20 μM) were set as controls. Cells grown in media containing an equivalent amount of DMSO without any drug served as blank control. After 12 h incubation, the cells were harvested and fixed overnight in cold 75% ethanol at 4 °C. After that, cells were washed again with pre-cold PBS and incubated with 100 μL RNase (100 μg/mL) at 37 °C for 1 h. Subsequently, cells were stained with 100 μL PI (100 μg/mL) for 15 min at 4 °C in dark, and subjected to FACS Calibur flow cytometry (BD Biosciences, Franklin Lakes, NJ, USA), to determine the percentage of cells in specific phase of the cell cycle (G1, S, and G2/M).

### 4.9. Cytoskeleton Destruction and Actin-Polymerization Study

To identify the destruction of cytoskeleton caused by CE + DTX, the cytoskeleton was stained and observed. B16 and MCF-7 cells were cultured in 35 mm glass bottom dishes in complete medium for 24 h attachment, then were exposed to CE + DTX combination solution (final concentration of CE and DTX were 5 and 10 μM, respectively) for 12 h at 37 °C under 5% CO_2_. Cells treated with CE solution (5 or 10 μM) and DTX solution (10 or 20 μM) were set as controls. Cells grown in media containing an equivalent amount of DMSO without any drug served as blank control. After being fixed with 4% paraformaldehyde in PBS, the cells were permeabilized with 0.1% Triton X-100 and subsequently treated with 3% bovine serum albumin (BSA)-PBS solution. Actin filament (F-actin) was stained with FITC-phallacidin (4 μg/mL) and β-tubulin was stained with Tubulin-Tracker Red (3 μg/mL), respectively, for 1 h at 37 °C. Then Hoechst 33342 staining was performed to stain the nucleus. Finally, the glass bottom dishes were examined and photographed under a fluorescence microscope (BX40, Olympus, Tokyo, Japan).

To further investigate the polymerization of actin caused by CE, the flow cytometry assay was employed. B16 and MCF-7 cells were treated with fresh culture media containing CE at concentration of 5 μM for 12, 24, and 36 h, respectively. Actin filament (F-actin) was stained with FITC-phallacidin (4 μg/mL). After that, cells were trypsinized and harvested. Then, the samples were measured by FACS Calibur flow cytometry (BD Biosciences, Franklin Lakes, NJ, USA).

### 4.10. In Vivo Antitumor Efficacy Evaluation

In order to verify the *in vivo* synergistic effects of CE combination, the *in vivo* antitumor efficacy evaluation was experimented. The antitumor effect of CE + DTX was evaluated in Kunming mice (18–22 g) inoculated with B16 melanoma cells (3 × 10^5^) by subcutaneously injection at the right axillary space. Treatments were started after 10–12 days of the implantation. The mice with tumor volume of ~100 mm^3^ were selected and this day was designated as “Day 0”. The mice were randomly assigned to 6 treatment groups: (1) 2.5 mg/kg CE; (2) 5 mg/kg CE; (3) 10 mg/kg DTX [36]; (4) 20 mg/kg DTX [43,44]; (5) CE + DTX (DTX: dosage of 10 mg/kg, CE: dosage of 2.5 mg/kg, final combination molar ratio of CE and DTX was 0.5:1); (6) normal saline (N.S). CE-solution was obtained by diluting DMSO stock solution with physiological saline while DTX-solution and CE + DTX-solution were obtained by diluting the Tween^®^-80-ethanol stock solution (Sinopharm Co., Ltd., Shanghai, China) with physiological saline. The mice were administered intravenously with the above-mentioned formulations once every three days for 21 days [43,44].

All mice were labeled, and the tumors were measured every three days with calipers during the period of study. The tumor volume was calculated by the formula:

(1) $$V=(W^{2}\times L)/2$$

where *W* is the tumor measurement at the widest point and *L* is the tumor dimension at the longest point. Each animal was weighed at the time of administration, so that the dosage could be adjusted to achieve the required dose (mg/kg) reported. After 21 days, the animals were sacrificed and the tumor mass was harvest and weighed.

### 4.11. Statistical Analysis

All studies were repeated a minimum of three times and measured at least in triplicate. Results were reported as means ± standard deviation SD. Statistical significance was analyzed using the Student’s *t*-test. Differences between experimental groups were considered significant when the *p*-value was less than 0.05 (*p* < 0.05).

## 5. Conclusions

This study made a comparatively comprehensive exploration on the potential application of CE in combination with anti-cancer drugs, making up for the deficiency of the previous research in the field of CE-based combination therapy. Meanwhile, the synergy mechanisms of CE + DTX were firstly elucidated. All these results in our study might provide a theoretical basis for CE combination and established a proof-of-concept that a rational combination of CE with DTX could generate synergistic effects on cancer treatments, which is highly promising for preclinical and clinical investigation to enhance therapeutic efficacy.

## Supplementary Information



## Figures and Tables

**Table 1.** Combination index (CI) values of CE + DTX with different combination molar ratio at 50% growth inhibition point on B16 and MCF-7 cells (*n* = 3).

CE + DTX		Combination molar ratio				
		0.25	0.5	1	2	4
**B16**	CI at IC50	0.49 ± 0.17	0.31 ± 0.13	0.47 ± 0.14	0.95 ± 0.26	1.08 ± 0.17
	Interpretation	synergism	strong synergism	synergism	additive effect	additive effect
**MCF-7**	CI at IC50	0.62 ± 0.13	0.48 ± 0.11	0.79 ± 0.25	1.17 ± 0.18	1.03 ± 0.19
	Interpretation	moderate synergism	synergism	moderate synergism	moderate antagonism	additive effect

Note: CI > 1.3: antagonism; CI 1.1–1.3: moderate antagonism; CI 0.9–1.1: additive effect; CI 0.8–0.9: slight synergism; CI 0.6–0.8: moderate synergism; CI 0.4–0.6: synergism; CI 0.2–0.4: strong synergism.
