# Supplementary material for: Synergistic Enhancement of Cancer Therapy Using a Combination of Ceramide and Docetaxel
Source: Int J Mol Sci. 2014 Mar 10;15(3):4201–20. doi: 10.3390/ijms15034201 (PMC3975392; doi:10.3390/ijms15034201)
Supplement: Supplementary file 1 [file ijms-15-04201-s001.pdf]

Supplementary Information

Figure S1. Cytoskeleton destruction effect of CE + DTX on B16 cells (n = 3). The blue color indicated the location of nuclei, the green color indicated the location of F-actin and the red color represented the β-tubulin. The white arrows indicate the stress fibers of F-actin. Scale bar, 200 μm.

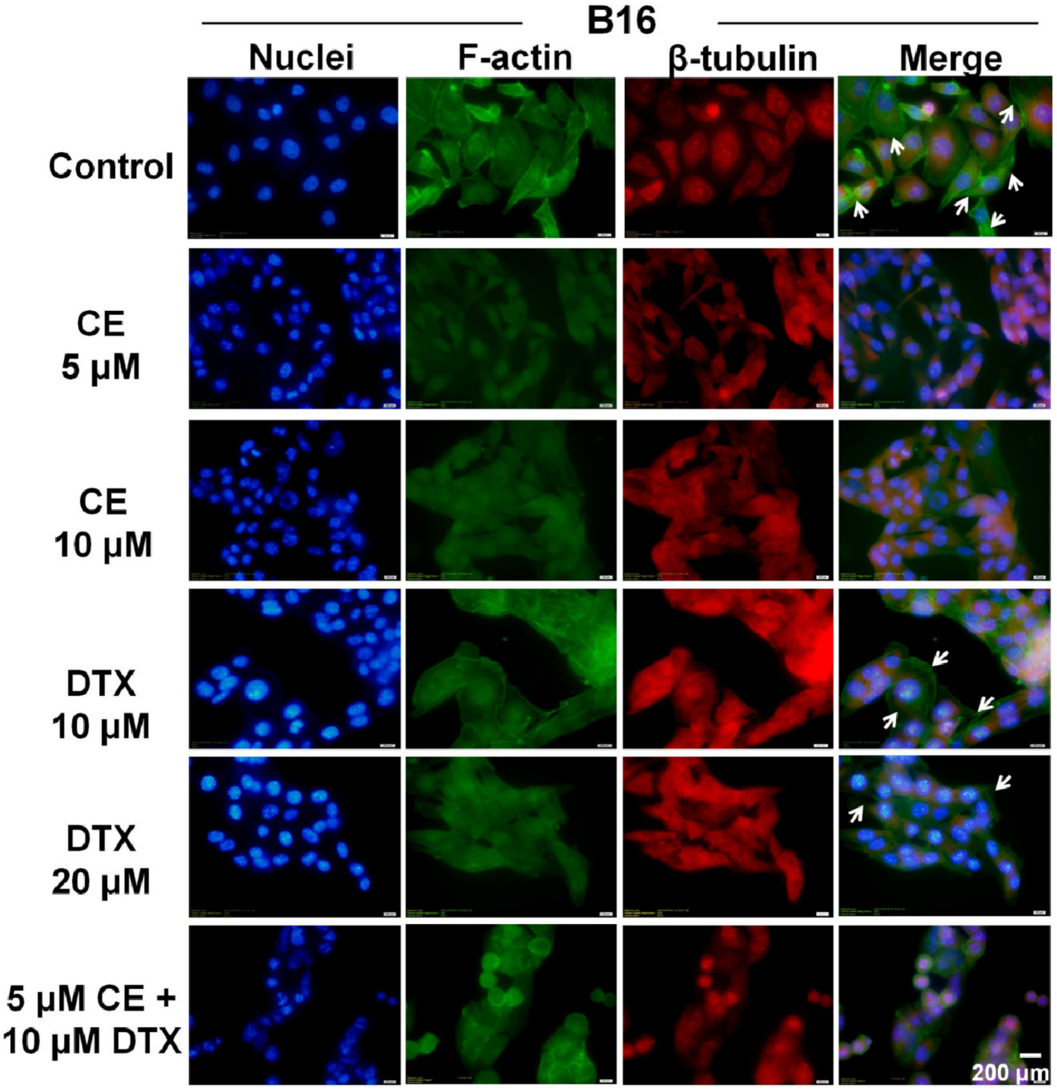

© 2014 by the authors; licensee MDPI, Basel, Switzerland. This article is an open access article distributed under the terms and conditions of the Creative Commons Attribution license (http://creativecommons.org/licenses/by/3.0/).
